# Advanced Smartphone-Based Sensing with Open-Source Task Automation

**DOI:** 10.3390/s18082456

**Published:** 2018-07-29

**Authors:** Maximilian Ueberham, Florian Schmidt, Uwe Schlink

**Affiliations:** 1 Department of Urban and Environmental Sociology, Helmholtz Centre for Environmental Research—UFZ, 04318 Leipzig, Germany; uwe.schlink@ufz.de; 2 LeanERA GmbH, 04109 Leipzig, Germany; florianschmidt2207@gmail.com

**Keywords:** smartphone sensors, personal exposure monitoring, task automation, participatory sensing, acoustic noise, geolocation, cycling

## Abstract

Smartphone-based sensing is becoming a convenient way to collect data in science, especially in environmental research. Recent studies that use smartphone sensing methods focus predominantly on single sensors that provide quantitative measurements. However, interdisciplinary projects call for study designs that connect both, quantitative and qualitative data gathered by smartphone sensors. Therefore, we present a novel open-source task automation solution and its evaluation in a personal exposure study with cyclists. We designed an automation script that advances the sensing process with regard to data collection, management and storage of acoustic noise, geolocation, light level, timestamp, and qualitative user perception. The benefits of this approach are highlighted based on data visualization and user handling evaluation. Even though the automation script is limited by the technical features of the smartphone and the quality of the sensor data, we conclude that task automation is a reliable and smart solution to integrate passive and active smartphone sensing methods that involve data processing and transfer. Such an application is a smart tool gathering data in population studies.

## 1. Introduction

Smartphones are nowadays well established and commonly used for private purposes [1]. Thousands of applications exist that support users in several ways, e.g., for navigation, fitness, messaging, or daily scheduling [2]. In comparison to that, environmental research is a relatively new area of smartphone application. Its usefulness has been shown in recent years as technological tools in numerous research areas, e.g., to capture notes, conversations, pictures, videos, or for remote monitoring [3,4]. Besides, the technological features of the smartphones become more important and new developments allow for increasingly specialized applications.

Thus, smartphones have recently been equipped with smaller and technically advanced sensors to monitor motion, position, temperature, humidity, light, air pressure, noise, or heart rate. These parameters reveal novel possibilities of signal processing for research purposes.

Today, there are already publications about smartphone sensing methods (SSMs) on topics of

- ecosystem services in geo- and citizen science [5,6,7],
- human activity, health, and well-being in medicine and sports science [8,9,10,11,12],
- urban microclimate in meteorology [13,14],
- air pollution and noise in geography [15,16,17],
- mobility and human movements in transport planning [18,19,20], and
- social interaction and behavior in social science [21,22,23].

Based on these publications SSMs can be differentiated in active and passive sensing tasks. Passive SSMs enable automated collection of smartphone generated data, for example, by the accelerometer, GPS data, and ambient noise levels. Active sensing tasks ask for an active user contribution such as taking a picture, tagging a place or entering text. The passive collection is more often used in quantitative and active collection in qualitative research.

A special case is citizen-science research that builds on different research subjects with the aim of involving non-professional scientists or the general public in projects to generate collective knowledge through co-production [5,24]. In this context, the field of participatory sensing is coming to the forefront of methods that are used [25]. The collective use of SSMs in participatory sensing empowers people to use their own smartphone to collect data on issues in their own interest, e.g., noise along near road, air pollution on the balcony, or heat stress at work [26,27,28].

The aforementioned publications have in common that they use either passive or active SSMs or focus just on single sensor signals. There is a lack of knowledge about the integrated use of multiple smartphone sensor signals with a combination of active and passive SSMs in scientific research. As interdisciplinary research projects tend to work on applied studies were both quantitative and qualitative data are collected, we see substantial needs to advance SSMs for this purpose. Task automation for smartphones could be one valuable solution. Automation applications for smartphones can access all internal and external sensors, as well as audiovisual features to perform user specific tasks. To the best of our knowledge, automation applications for smartphones have not been used or applied in environmental research and participatory sensing to date. Environmental science is a research area where SSMs offer tremendous potential for mobile data collection and monitoring [4,29]. So far, they are used within the coder community to design task applications that can simplify everyday tasks [30].

Therefore, our aim was to investigate whether smartphone software can be used to manage multiple sensor signals for the integration of passive and active sensing and to use this data for spatio-temporal context analysis. We designed a script for task automation that stores quantitative records of noise and light-level together with timestamp, geolocation, and a qualitative user feedback.

The applicability has been validated through the visualization of measurements and a rating of the users’ handling experiences. We applied the smartphone application in a study about personal exposure of cyclists because they are exposed to several environmental stressors in daily life, like acoustic noise, air pollution, and heat [31,32]. Assessing this everyday exposure is a major methodological challenge as people are moving in space. For our case study, we took the importance of end-user comfort very seriously when designing the task application. As participants in exposure studies are usually from the general public and they have no special technical knowledge, it is important to keep SSMs as simple as possible to guarantee an unimpeded study and to keep participants highly motivated.

We also discuss the application possibilities for environmental research in general and conclude with recommendations for studies that consider using task automation with passive and active SSMs.

## 2. Materials and Methods

### 2.1. Device Sensors and Task Application

**Figure 1:**
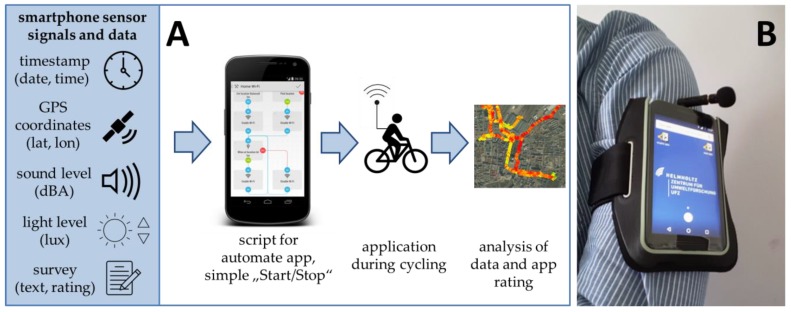
Study design (**A**). Smartphone with external microphone attached to arm (**B**).

The basic equipment included 15 smartphones (Motorola G3, Android 6.0) that are available for the participatory sensing study with cyclists. As we were interested in the spatio-temporal context of the noise exposure, the location of the cyclists was of major importance. Therefore, we integrated geolocation via GPS coordinates and used the open source application “GPS Logger” (https://code.mendhak.com/gpslogger/). It was furthermore important to control for (a) outdoor application and (b) wearing compliance. We instructed the participants to use the smartphone being attached to the upper arm only outdoors while cycling. Light levels above a predefined threshold indicate whether the person was in an outdoor environment or not. Finally, an active sensing part was integrated in form of a short survey that appeared on the screen and asked for the user’s feedback. We asked for (a) the purpose of the trip (answer as free text field: e.g., work, home, leisure), (b) type of route choice (answer options: habitual, unusual), (c) taken detours (answer options: yes, no), and (d) rating of perceived exposure to noise on this route (answer on a rating scale: 1 low–5 high, Figure 1). The timestamp of the smartphone was the record reference synchronizing all of the sensor signals. All sensor signals that were utilized are listed in Table 1.

We focused on the concept of automated task applications, as they can automate various tasks on the smartphone. They capture the sensor signals from the device and can combine, process, and store the values based on commands, time, or location. The main requirement for the application was the ease-of-use aspect. Therefore, we worked with a “start and stop widget” on the smartphone’s homescreen. In addition, we integrated a task for automatic data transfer to a database server, to ensure immediate access after collection.

We finally have chosen the open-source task application *Automate* (http://llamalab.com/automate/) to design a script for the management of the sensor signals. *Automate* is a basic software that operates using flowcharts (“flows”) of tiles. Each tile will perform a single task and a group of tiles is named “fiber”. All of the fibers together form the flow (a script code) that ends up in the intended output (e.g., value, table). A basic principle to connect the tiles are logic operations (e.g., IF, AND, OR). We used the Automate Version 1.7.1 that is available at the Android Google Play Store.

Except for acoustic noise, all of the sensors were internal features of the smartphone. The sound level was detected by an external microphone with foam windscreen (Edutige ETM-001), as we noticed that the internal microphone would be covered by the bracelet during application, thus causing errors.

### 2.2. Application Evaluation

The final programmed application was evaluated in three ways: based on (1) the operability of the script and correct data output, (2) geographic visual proof of the data, and (3) the ease-of-use rating of cyclists that participated in the exposure study.

The correct working script stored all merged sensor signal data of the recorded bike trip in a comma separated file (csv) on the local smartphone memory. Additionally, the file was automatically transferred to a database on a cloud server in near real time.

The visual and geographical proof of the data output is of high relevance as data was needed for further spatio-temporal analysis in the exposure study project. It is of importance that the cycling tracks (GPS) are correctly represented and merged with the other sensor data.

The ease-of-use rating of the application was performed after the exposure study. Therefore, 66 cyclists used the same study smartphone with the installed automation application for one week during daily cycling in the study period from June to September 2017. The study area was the City of Leipzig, Germany. All of the participants signed written informed consent. The study design was approved by the ethics committee of the Leipzig University (No. 191/17-ek).

The smartphones had mobile internet connection to allow for direct data transfer to the cloud server. Before starting the bike trip the users had to press just a start-widget on the smartphone’s homescreen and to attach the device on the left arm. After finishing the bike trip, users simply pressed the stop-widget and answered the appearing questions related to the trip and exposure perception. The final ease-of-use evaluation of the device handling was done via an online survey that the users filled after their study participation. They rated the handling of the application (1 very easy–5 very complicated) and the wearing comfort of the smartphone (1 very high–5 very low).

## 3. Results

### 3.1. Skript Programming

The developed script for *Automate* was able to retrieve the desired sensor signals and store them in a predetermined interval (in our case 2 s) with a timestamp in a table. In addition, a text and scoring query was successfully integrated, which makes it possible to retrieve additional information from the user. We inquired about the purpose of the cycle route, possible detours, and the subjectively perceived exposure to noise.

**Figure 2:**
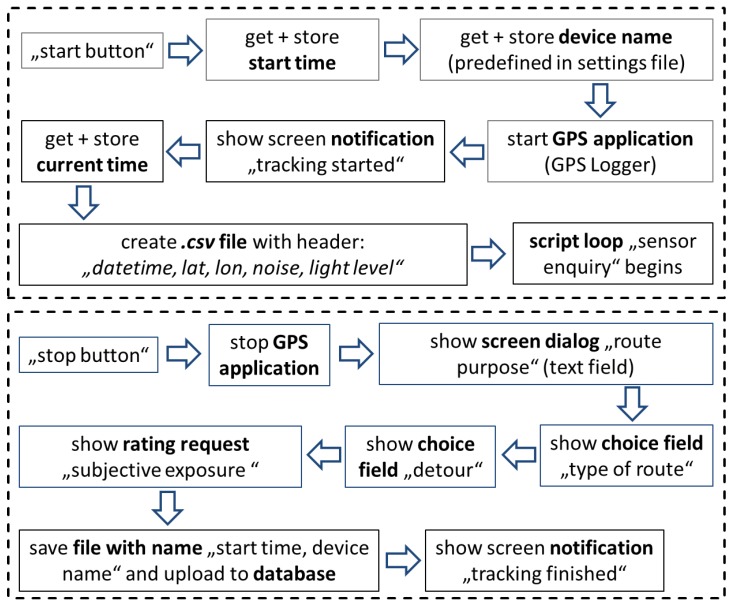
Basic script flowchart.

Therefore, we designed two flowcharts with the software (Figure 2). One for “start track” and one for “stop track”. The complete and detailed flowchart scripts can be found in Figure S1.

When the users pressed the start-widget, the application recorded the current timestamp and the name of the smartphone, what was later used to name the target file. The device name is predefined by a separate text file, which is stored in the mobile phone memory. As soon as satellite signals are connected, the measurement loop starts. This will be reported to the user with a screen notification. At the same time, the target csv-file is created and all of the predefined sensor parameters are stored in intervals using the current timestamp.

Arrived at his destination, the user stops the measurement pressing the stop-widget. Subsequently, the GPS application stops tracking and the screen immediately display queries about the purpose of the cycle route, possible detours, and subjective perceived exposure (see Figure S2 for exemplified screenshots). This active sensing data is stored in a separate csv-file. Finally, the notification appears that the measurement has been completed.

### 3.2. Data Output and Visualization

The operability of the script was evaluated based on the successful storage of a csv-file in our predefined server database immediately after stopping the application. We obtained a spreadsheet with the header “datetime, latitude, longitude, noise, light level”. A 2 s recording interval was selected to capture small-scale differences of the noise intensity. This results in approximately 6 m distance between each measurement (average speed 20 km/h). The interval can be adjusted in the script.

While testing the script output, we made some adjustments to improve the data format. For example, in a first version we had just one column for the GPS coordinates. The final version stores latitude and longitude in separate columns with the advantage of easier post processing for visualization with GI-software (e.g., Google Maps, ArcGIS).

**Figure 3:**
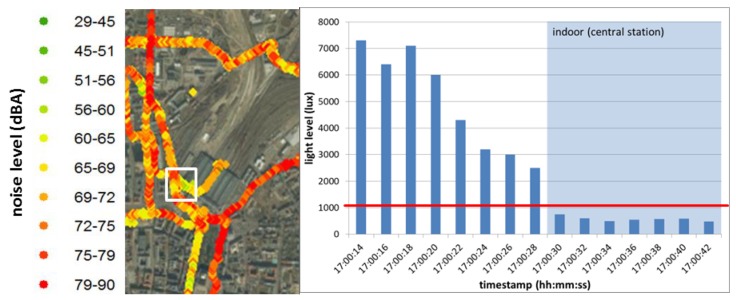
GIS-visualization of noise (dBA) data recorded within the white framed area (**left**) and the corresponding light levels for this timeframe with threshold of 1000 lux in red (**right**).

As our final aim was the visualization of the data to reveal personal spatio-temporal exposure to environmental stressors (in this case acoustic noise), we imported the original spreadsheet into ArcGIS. The resulting map shows a segment of a study participant’s cycling track (Figure 3).

The map on the left hand side shows the noise levels (29–90 dBA) that were registered at the locations (GPS). Here, we plotted the raw data without outlier removal to highlight the usefulness of the recorded light level data. The light sensor, which actually controls the brightness of the screen, can provide information about whether the device is indoors (<1000 lux) or outdoors (>1000 lux). In our study, we benefit from this data as exclusion criteria for indoor recordings, as we are just interested in the cyclists’ outdoor exposure. An exemplified timeframe in which the cyclist went into a building is represented by the white frame on the map (Figure 3, left). The corresponding light level is plotted on the graph (Figure 3, right). The GPS-track on the map reveals that the study participant enters the central station at 17:00:30 as the light level drops < 1000 lux. This prevents mismeasurements in case the user did not stop the application directly after the end of the cycling trip. Besides this incorrectness, the application was sometimes started before the bike trip was started (e.g., when leaving the apartment). In another case with a constant low light level outdoors, the participant did not meet the wearing compliance (smartphone worn in breast pocket, not with bracelet). In all cases, we excluded the recorded data with low light level from further analyses.

### 3.3. Rating of Handling and Wear Comfort

During the pilot study with cyclists, we already got direct oral feedback that was generally positive when we picked up the smartphones. The application was used by each cyclist 12 times (12 routes) on average. 59 cyclists (55% female) filled the feedback questionnaire online afterwards. Therefore, the ease-of-use evaluation is based on about 708 user-application interactions.

**Figure 4:**
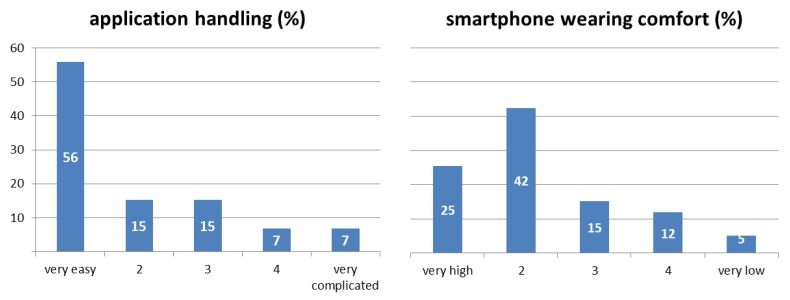
Results of ease-of-use rating for the application/smartphone (*n* = 59).

The results shown in Figure 4 indicate that the majority of participants (71%) rated the handling of the application as easy or very easy (median = 1). Whereas, the wearing comfort was rated slightly lower with 67% for very high and high (median = 2). This implies that the application was in general easy to handle for the cyclists with potential in wearing comfort improvements. For both ratings, we found no significant differences in the mean values for age and gender (U-Test > 0.05).

## 4. Discussion

The developed *Automate-*script for smartphone task automation demonstrated that multiple sensor signals can be integrated with time and geolocation to collect data in a personal exposure study with cyclists. The resulting data can be used for further spatial analysis and contribute to an improved understanding of the spatio-temporal exposure to environmental stressors. The greatest advantage of our task automation is the integration of active sensing through the request of notifications that enable user feedback after tracking. Thereby, it is possible to compare this subjective data with the objective data from passive sensing in relation to the same timeframe. This is of special interest for studies were complex spatial problems are investigated that need to consider quantitative and qualitative aspects.

With our script, we showed that task automation is a reliable SSM that has potential to integrate multiple sensor signals. Many combinations of sensor signals are possible and our example to use the light level has shown that the original purpose of the sensor can be utilized as another helpful indicator in a research project. The software *Automate* offers over 300 different task commands to access, combine, store sensor signals, and applications in the smartphone. Furthermore, external sensors can be connected to the device via WLAN, Bluetooth, or NFC.

Smartphones can be used in many research areas as an advanced tool for data management due to their penetration in society and ongoing evolving technology [4]. This technological possibility paves the way to approach several applied research questions among others in natural science, geography, social science, citizen science, public health, and exposure science.

Beside the relevance of our project to advance SSMs, we emphasize that it is always necessary to consider the accuracy of smartphone sensors. It was not the aim of this article to describe the accuracy of the recorded data, but several articles already confirmed the good sensor performance of smartphones in relation to GPS and noise [33]. Furthermore, the comparability of the results depends on the smartphone device model. We have used the same smartphone in our study with Android OS. Further research should check data output from different device brands and operation systems. It is clear that the energy consumption of the smartphone is much higher during the sensing process, but varies based on the kind and amount of sensor signals. Wang et al. however proposed an algorithm to manage energy consumption more efficiently [34].

The article highlighted the process of data collection and showcased a possible visualization scenario. For data storage we used a server database. We see further development needs in the integration of automated cleansing of the data within the database server to forward the data to a Web-GIS application for direct visualization. This possibility would allow for interactive visual user feedback either on a website or on the smartphone. This kind of data processing is already established in the context of different GPS-tracking applications where alternative routing is suggested to the user. The recording, analyzing, mapping, and visualization of multiple smartphone sensor data, including an active user interaction, still needs further experiments and is an emerging field of research [22].

## 5. Conclusions

In this paper, we presented a script to automate smartphone task commands for application with sensors in the context of participatory personal exposure studies. The novelty of our script is the integration of active and passive SSMs by means of utilizing time, geolocation, noise level, light level, and a digital questionnaire. An evaluation of this application by cyclists revealed the reliable handling and ease-of-use. According to the trend that data should be available in near real-time, we used a wireless database server storage solution in our script. In future, the real-time data transfer might be integrated and run in parallel to the sensing process; this however depends on the quality and speed of the user’s mobile internet signal that can be poor in rural areas. When considering the limitations of the technology, automated task applications for smartphones are a promising way to advance empirical research involving real-world data collection, especially in the area of environmental science.

## Figures and Tables

**Table 1:** Sensor signals and value range.

Proxy	Timestamp	GPS, Latitude	GPS, Longitude	Sound Level	Light Level	User Feedback
**SSM**	passive	passive	passive	passive	passive	active
**value unit**	dd.mm.yyyy hh:mm:ss	decimal degrees	decimal degrees	decibel A-weighting (dBA)	lux	text, ordinal rating
**range**	0–24 h	51.25–51.40	12.24–12.51	30–90	0–60.000	1–5
**sensor**	internal clock	internal GPS, WLAN, GSM		external microphone	internal light sensor	screen

## Supplementary Materials

The following are available online at http://www.mdpi.com/1424-8220/18/8/2456/s1, Figure S1: Automate Flowchart Script, Figure S2: Smartphone screenshots.
