# Supplementary material for: Advanced Smartphone-Based Sensing with Open-Source Task Automation
Source: Sensors (Basel). 2018 Jul 29;18(8):2456. doi: 10.3390/s18082456 (PMC6111588; doi:10.3390/s18082456)
Supplement: Supplementary file 1 [file sensors-18-02456-s001.pdf]

Supporting information for article:

Advanced smartphone-based sensing with open-source task automation

Maximilian Ueberham [1*], Florian Schmidt [2] and Uwe Schlink [1,]

[1] Department of Urban and Environmental Sociology, Helmholtz Centre for Environmental Research – UFZ, , 04318 Leipzig, Germany; E-Mails: maximilian.ueberham@ufz.de; uwe.schlink@ufz.de
[2] LeanERA GmbH, 04109 Leipzig, Germany; florianschmidt2207@gmail.com
* Correspondence: maximilian.ueberham@ufz.de; Tel.: +49-341-235-1740

2 pages with 1 figure

Figure SI-1: Flowchart of script for task automation
Figure SI-2: Smartphone screenshots

Figure SI-1: Script flowchart (Start-Flow and Stop-Flow)

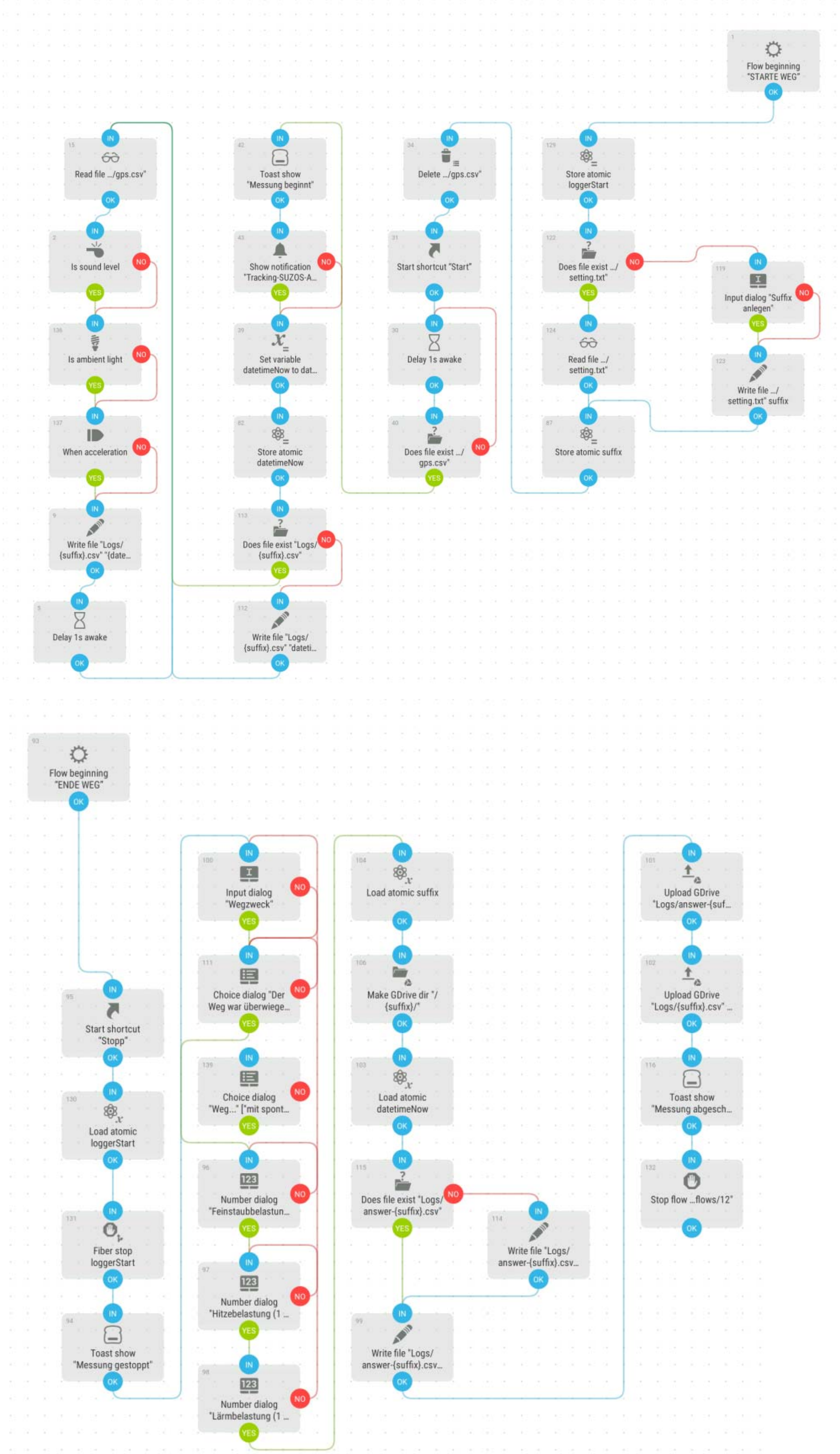

Figure SI-2: Smartphone screenshots (a: home screen with start and stop widget, b: screen dialog “route purpose”, c: rating request “perceived noise exposure”)

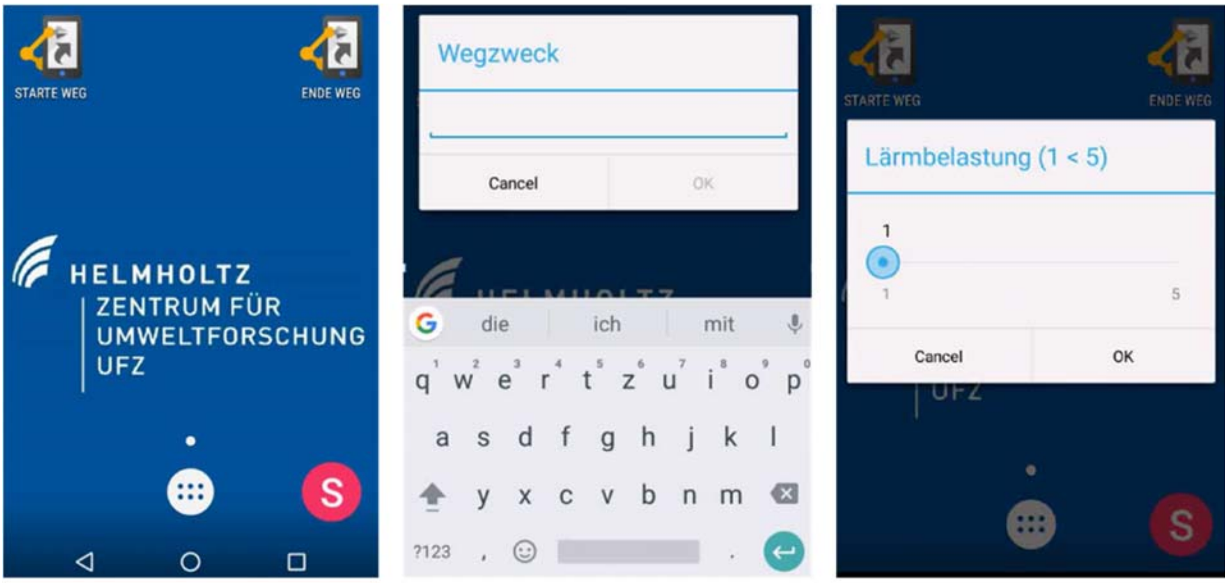

a b c
